# Accuracy and reproducibility of two-dimensional computed tomography-based positioning of femoral component rotational alignment in preoperative planning for total knee arthroplasty

**DOI:** 10.1186/s13018-023-04466-1

**Published:** 2023-12-14

**Authors:** Kun Liu, Yuandong Liu, Zongqing Fan, Donglin Fu

**Affiliations:** Fuyang People’s Hospital, Anhui Medical University, Fuyang, 236000 Anhui Province China

**Keywords:** Total knee arthroplasty, Preoperative planning, Rotational alignment, Posterior condylar angle, Two-dimensional(2D) measurement, Three-dimensional(3D) measurement

## Abstract

**Background:**

Poor rotation of the femoral component in total knee arthroplasty (TKA) can result in various postoperative complications, underscoring the critical importance of preoperative planning.

**Purpose:**

To improve the accuracy of femoral component positioning during TKA, this study compared the accuracy and repeatability of different two-dimensional (2D) computed tomography (CT) measurement methods for measuring the posterior condylar angle (PCA) in preoperative TKA planning.

**Methods:**

A retrospective analysis was conducted on 75 patients (150 knees) who underwent bilateral lower extremity computed tomography angiography (CTA) at Fuyang People's Hospital from January 2021 to July 2021. Three different methods were used to measure the PCA based on 2D CT images (axial CT slices) and three-dimensional(3D) models (femoral models reconstructed from CT data) in this study. Method 1: Single-plane 2D CT measurement, measuring PCA in the most obvious single-plane CT slice of the surgical transepicondylar axis (sTEA); Method 2: multi-plane 2D CT measurement, identifying and locating anatomical landmarks in multiple 2D CT slices and measuring PCA; Method 3: 3D model measurement, measuring PCA in the reconstructed femur 3D model. Compare the differences in PCA measurements between the three measurement methods. A positive PCA measurement was recorded when the sTEA was externally rotated relative to the posterior condylar line (PCL). Any difference exceeding 3° between the PCA measurement in the 2D CT and the PCA reference value in the 3D model was classified as an outlier. The intraclass correlation coefficient (ICC) and Bland–Altman method were utilized to assess the intra- and inter-observer reproducibility of the three measurement methods.

**Results:**

The PCA measurement in the single-plane 2D CT was 1.91 ± 1.94°, with a measurement error of − 1.22 ± 1.32° and 12.7% of outlier values. In the multi-plane 2D CT, the PCA measurement was 2.96 ± 1.68°, with a measurement error of -0.15 ± 0.91° and 6.0% of outlier values. The PCA measurement in the 3D model was 3.12 ± 1.69°. The PCA measurement in single-plane 2D CT was notably smaller than that in multi-plane 2D CT and 3D models, with no significant difference between the latter two. The multi-plane 2D CT showed significantly lower measurement error and outlier values than the single-plane 2D CT. All three PCA measurement methods exhibited high reproducibility (ICC: 0.93 ~ 0.97).

**Conclusions:**

Using of multi-plane 2D CT for measuring PCA in preoperative planning of TKA has high reproducibility and accuracy, with fewer outlier values. We recommend preoperative measurement of PCA using muti-plane 2D CT to improve the accuracy of positioning the femoral component rotational alignment during surgery.

## Introduction

Total knee arthroplasty (TKA) is the most effective treatment for end-stage knee osteoarthritis [1], providing significant pain relief and improvement in knee joint function, with an implant survival rate of over 90% for 15 years [2]. However, up to 20% of patients remain dissatisfied with the clinical outcomes [3], mainly due to poor functional recovery and persistent pain in the knee joint after surgery [4]. The rotational alignment of the femoral component on the axis plane is a critical factor affecting the clinical efficacy of TKA [5]. Poor rotation of the femoral component can lead to abnormal gait, unbalanced flexion gap, and poor patellar tracking [6], which can result in complications such as patellar dislocation and subluxation, unstable knee flexion, and anterior knee pain [4]. Currently, the main methods for determining the rotational axis of the femoral component are measured resection and gap balancing techniques [7]. The measured resection technique determines the rotational alignment of the femoral component by referencing anatomical landmarks of the distal femur to establish the location of the posterior femoral condyles cutting line [8]. Common anatomical landmarks include the surgical transepicondylar axis (sTEA), anatomical transepicondylar axis (aTEA), posterior condylar line (PCL), and Whiteside's line [9]. The sTEA is a line connecting the most prominent point on the lateral femoral epicondyle to the medial epicondyle sulcus. It is considered the physiological center axis of knee flexion and extension [10]. The PCL is a line connecting the lowest points on the medial and lateral posterior condyles of the femur. It is currently used as the reference axis for most knee arthroplasty instruments. The posterior condylar angle (PCA) is the angle between the sTEA and the PCL. Berger et al. [11] initially measured the sTEA in normal adult femoral specimens and found it to be externally rotated relative to the PCL by approximately 3°. Therefore, it is currently common practice to position the femoral component rotationally relative to the PCL with a 3° external rotation. However, the PCA measured by Berger et al. was based on normal femoral specimens without osteoarthritis and skeletal deformities, and it has been shown that when degenerative changes occur in the knee joint, the PCA varies with changes in the skeletal morphology of the distal femur [12]. In addition, research has also shown that PCA can differ according to age, gender, and race [13, 14]. Therefore, positioning the femoral prosthesis rotational alignment relative to PCL external rotation at 3° can lead to significant errors and is not accurate. In addition, the medial epicondylar sulcus is difficult to identify and locate intraoperatively in some individuals due to anatomic variation in the distal femur and soft tissue coverage [15]. Therefore, adequate preoperative planning is essential to achieve accurate positioning of the femoral component. In the preoperative planning of TKA, computed tomography (CT) is commonly used to assist in intraoperative femoral component rotation positioning by measuring PCA on an axial single-plane CT slice of the femur. This two-dimensional (2D) measurement method is easy to perform does not require additional specialized techniques, and has been shown to improve the accuracy of the femoral component rotational alignment [16, 17]. However, the four anatomical landmarks used to measure the PCA, including the most prominent point of the lateral femoral epicondyle, the sulcus of the medial femoral epicondyle, and the lowest points of the medial and lateral posterior condyles, may not be located on the same CT slice. As a result, there may be some level of error when measuring the PCA on a single-plane CT slice [18]. To date, no research has proposed a more precise 2D measurement method for PCA in preoperative planning for TKA. A 3-dimensional (3D) model of the femur reconstructed from CT data allows for detailed and precise preoperative planning. Currently, the accuracy of locating the femoral prosthesis rotational alignment based on 3D measurements has been recognized by many studies [19–21]. However, 3D measurement is a complex operation that requires additional specialized techniques. Therefore, it is necessary to explore an accurate and reliable 2D measurement method for PCA.

Objectives of this study: to measure PCA using two 2D as well as 3D measurement methods and evaluate their reproducibility; to compare the differences in PCA measurements obtained from two 2D and 3D measurement methods, and to evaluate the accuracy of PCA measured by the two 2D measurement methods. To provide new ideas as well as references for preoperative planning of femoral component rotational alignment in clinical TKA.

## Materials and methods

### Selection of subjects

This study was approved by the Ethics Review Board of Fuyang People's Hospital (IRB: [2022]79) and exempted from the requirement for informed consent. A retrospective analysis was conducted on 75 participants (150 knees) who underwent bilateral lower extremity computed tomography angiography(CTA) examinations at Fuyang People's Hospital between January 2021 and July 2021. Of the participants, 50 were male and 25 were female, with an average age of 70.05 ± 12.34 years. A total of 128 knees showed signs of degenerative osteoarthritis, while 22 knees showed no obvious signs of degenerative osteoarthritis.

Inclusion criteria: age 18 years and older; scanning range includes the complete femur. Exclusion criteria: surgical history affecting the localization of distal femoral anatomical landmarks and mechanical axis of femur; post-traumatic arthritis, rheumatoid arthritis; poor positioning of the lower extremities that do not meet the normal scanning conditions; poor CT image quality affecting measurement; unidentifiable medial epicondyle sulcus due to osteophytes or deformities, which affects the measurement of PCA.

### Bilateral lower extremity CTA examination

**Fig. 1:**
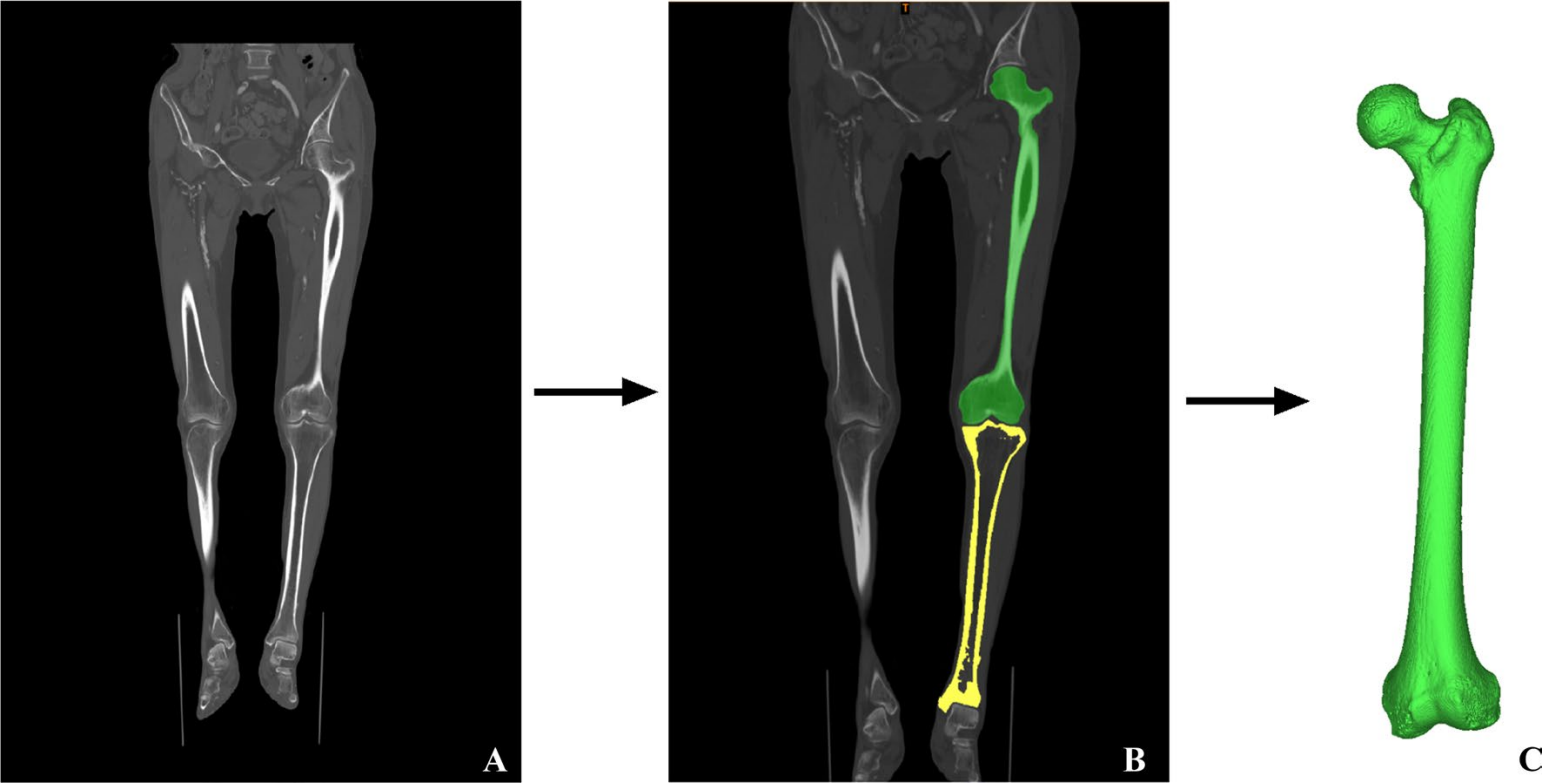
Extraction of femoral structure and establishment of the 3D model in Mimics software. Figure A: coronal view of bilateral lower extremities. Figure B: extracted and filled femoral structure. Figure C: reconstructed 3D model of the femur

The participants were positioned supine with bilateral lower extremity rotated to a neutral position, toes and patella pointing upwards, and both knees extended as much as possible. Bilateral lower extremity scanning was performed using a 64-slice multidetector CT scanner (Siemens Healthineers, Munich, Germany), with the scanning direction parallel to the mechanical axis of the femur. Scan parameters: slice thickness of 1.5 mm, voltage of 100kv, and tube current of 140 mA. Firstly, we measured the Hip-Knee-Ankle (HKA) angle between the mechanical axes of the femur and tibia in the coronal plane, using supine CT imaging. Subsequently, the scanned images were stored in the Digital Imaging and Communications in Medicine (DICOM) format. The collected DICOM data were imported into Mimics software (19.0, Materialise, Leuven, Belgium), and the target structure was extracted and filled to reconstruct the 3D model of the femur (Fig. 1).

### 2D measurements of PCA

#### Single-plane 2D CT measurement of PCA

**Fig. 2:**
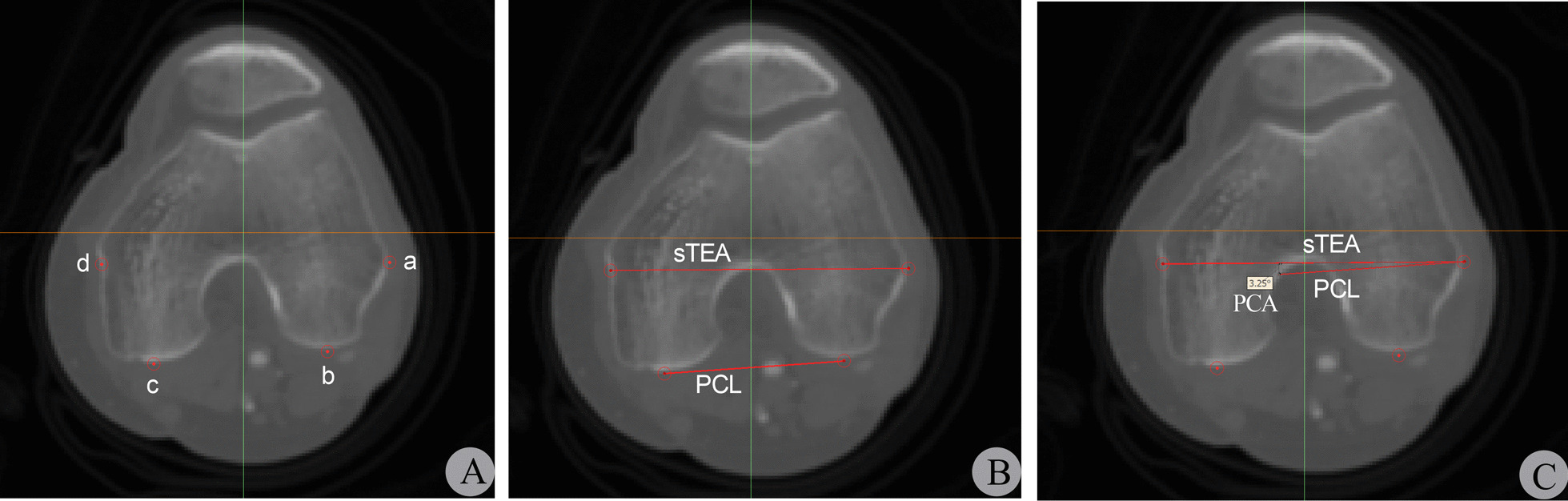
Localization of femoral distal anatomical landmarks and measurement of PCA in a single-plane 2D CT slice. Figure A: Localization of femoral distal anatomical landmarks. a: the most prominent point of the lateral epicondyle; b: the lowest point on the lateral posterior condyle; c: the lowest point on the medial posterior condyle; d: the medial epicondyle sulcus. Figure B-C: Localization of sTEA and PCL and measurement of PCA

The patient's bilateral lower extremity CTA data was imported into Mimics software in DICOM format. The femoral axial view frame was selected, and the CT slice with the most obvious sTEA was identified. The most prominent points of the lateral epicondyle and the medial epicondyle sulcus were clearly identifiable on this slice. The most prominent points of the lateral epicondyle, medial epicondyle sulcus, and the lowest points of the medial and lateral posterior condyles were marked on the selected slice. The angle of PCA was measured between sTEA and PCL (Fig. 2).

#### Multi-plane 2D CT measurement of PCA

**Fig. 3:**
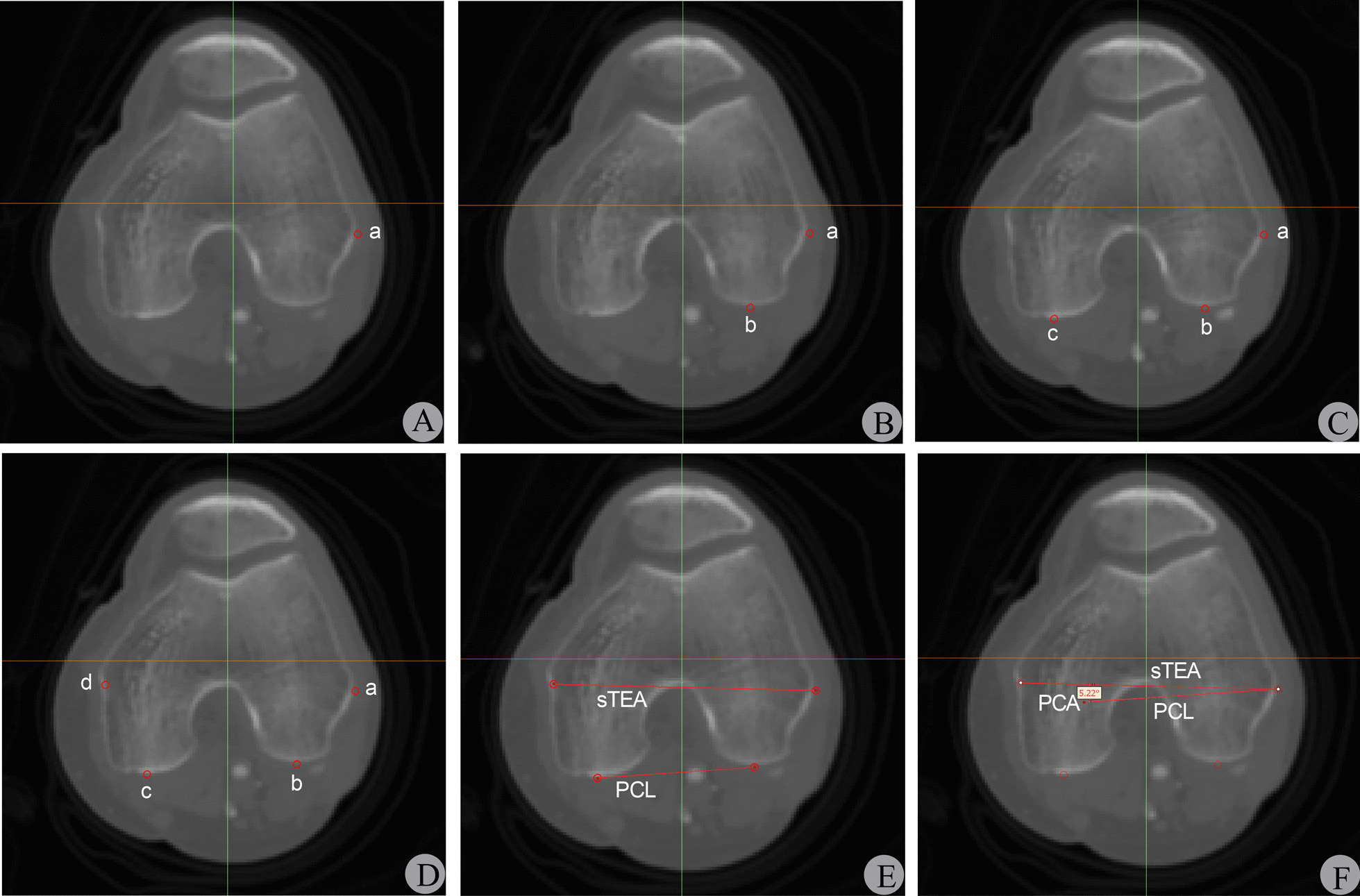
Localization of femoral distal anatomical landmarks and measurement of PCA in multi-plane 2D CT slices. Figure A-D: Localization of femoral distal anatomical landmarks. a-d correspond to the red cylindrical markers placed on the four most obvious points of the most prominent lateral femoral condyle, the lowest points on the lateral and medial posterior condyles, and the medial condyle sulcus, respectively. Figure E–F: Determination of sTEA and PCL and measurement of PCA

The CTA data were imported into Mimics software in DICOM format. The femoral axial view frame was selected, and the femoral distal CT scan plane was switched to identify the four CT slices with the most obvious points of the lateral epicondyle condyle (a), the lowest points on the lateral and medial posterior condyles (b, c), and the medial epicondyle sulcus(d). The most obvious anatomical landmarks on each of the four CT slices were marked with a red cylindrical marker (2 mm in diameter and perpendicular to the CT slice), and the markers' positions did not change with the plane switching. Finally, sTEA and PCL were drawn based on the four marked points, and the PCA was measured between the two lines (Fig. 3).

### 3D measurement of PCA

**Fig. 4:**
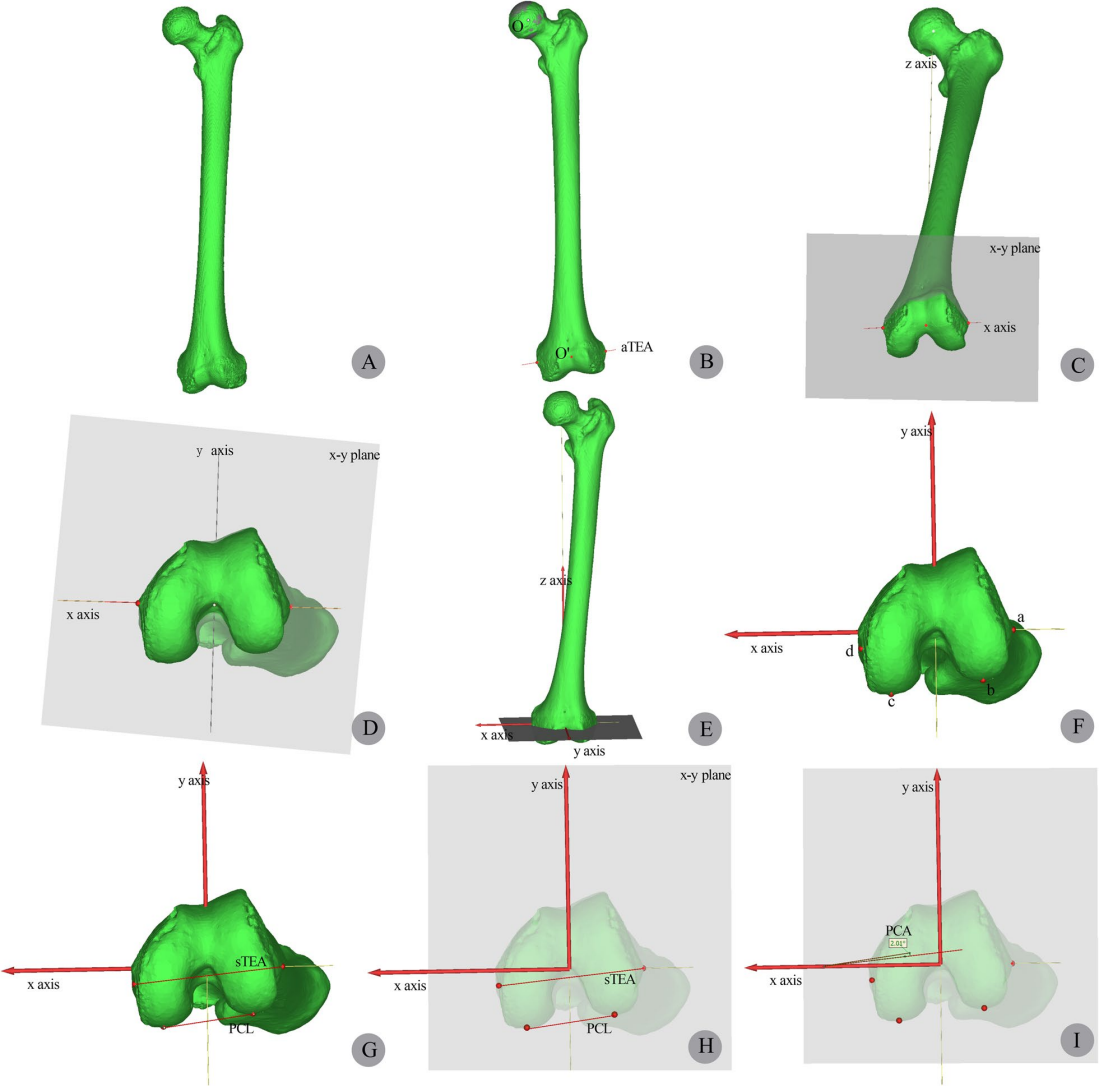
Establishment of the coordinate system and measurement of PCA in the 3D femoral model. Figure A: Reconstructed 3D femoral model. Figure B: Positioning of the hip joint center (O) and the knee joint center (O'). Figure C-D: Determination of the X, Y, and Z axes and the X–Y plane. Figure F: Localization of anatomical landmarks of the distal femur, with a ~ d indicating the most prominent point on the lateral epicondyle, the lowest points on the lateral and medial posterior condyles, and the medial epicondyle sulcus, respectively. Figure G: Localization of sTEA and PCL in the 3D model. Figure H-I: Measurement of PCA in the X–Y plane

A 3D model of the femur was reconstructed based on the CTA data using Mimics software, and 3D measurement were performed in the femoral model. We established a 3D coordinate system for the femoral model based on previous studies [22]. A sphere was selected to fit the femoral head, and its center was defined as the center of the hip joint. aTEA was defined as the line connecting the most prominent points on the medial and lateral femoral epicondyles, and its midpoint was defined as the center of the knee joint. The Z-axis was defined as the line connecting the center of the hip joint to the center of the knee joint along the femoral mechanical axis. The X–Y plane was defined as the plane perpendicular to the Z-axis at the center of the knee joint. The X-axis was defined as the projection of aTEA onto the X–Y plane, and the Y-axis was defined as the vertical line to the X–Z plane at the center of the knee joint. After locating bony landmarks in the femoral model, sTEA and PCL were drawn, and they were projected onto the X–Y plane. PCA was measured in the X–Y plane, with a positive value indicating external rotation of sTEA relative to PCL and a negative value indicating internal rotation (Fig. 4).

## Accuracy and reproducibility assessment of PCA measurements

We compared the differences in PCA measurements between 2D CT and 3D models, as well as the errors in two different 2D CT measurement methods, to evaluate the accuracy of different 2D CT measurements. Intraclass correlation coefficient (ICC) and Bland–Altman analysis were used to assess the intra- and inter-observer repeatability of 2D CT and 3D model measurements. All PCA measurements were performed by two independent researchers (KL and YDL), and measurements were repeated by the same researcher (KL) after a two-week interval. The difference between the PCA measurements obtained by the 2D CT methods and those obtained from the 3D model was defined as the measurement error. An error exceeding 3° was classified as an outlier, according to previous studies [12].

## Statistical analysis

Statistical analysis was performed using SPSS software (version 25.0, IBM, New York, USA). For continuous variables that followed a normal or approximately normal distribution, the mean and standard deviation ($\overline{x }$±$S$) were used for description, while categorical variables were presented as frequencies (%). The overall comparison of PCA measurements obtained by different methods was conducted using repeated measures analysis of variance (ANOVA), and pairwise comparisons were performed using Bonferroni correction. The paired *t*-test was used to compare the errors in PCA measurements obtained by the two 2D CT methods, and the chi-square test was used to compare the distribution of outliers between the two methods. The intra- and inter-observer repeatability was evaluated using intraclass correlation coefficient (ICC) and Bland–Altman analysis. ICC values less than 0.4 were considered poor consistency, values between 0.4 and 0.75 indicated moderate consistency, and values greater than 0.75 indicated high consistency [23]. Bland–Altman analysis evaluation criteria: more than 95% of the difference between the two measurements lies within the consistency limits (95% distribution range of the difference between the two measurements) [24]. *P* < 0.05 was considered statistically significant. The sample size was calculated using G*Power software [25](version 3.1, Heinrich-Heine-Universität Düsseldorf, Düsseldorf, Germany). For the repeated measures ANOVA, a two-tailed alpha of 0.05 was used, with a power of 95% and an effect size of 0.18. The results indicated that a total of 90 cases were required for the study, and a sufficient number of 150 cases were included in the analysis. Therefore, the sample size was considered reliable.

## Results

### General information about the subjects

This study included 75 subjects (150 knees) (all are Han Chinese) with a mean age of 70.05 ± 12.34 years (ranging from 42 to 94 years), of whom 50 were male and 25 were female. Among the participants, 128 knees showed signs of degenerative osteoarthritis. The HKA angle of the participants included in this study was 175.87 ± 4.48°, with 175.20 ± 4.03° (91.3%) for varus knees and 183 ± 2.61° (8.7%) for valgus knees. In addition, the PCA was 1.94 ± 1.89° for males and 1.84 ± 2.05° for females. PCA with osteoarthritis was 1.80 ± 2.52° and without osteoarthritis was 2.51 ± 1.35°.

### Comparison of PCA measurements between two 2D CT and 3D model measurement methods

**Fig. 5:**
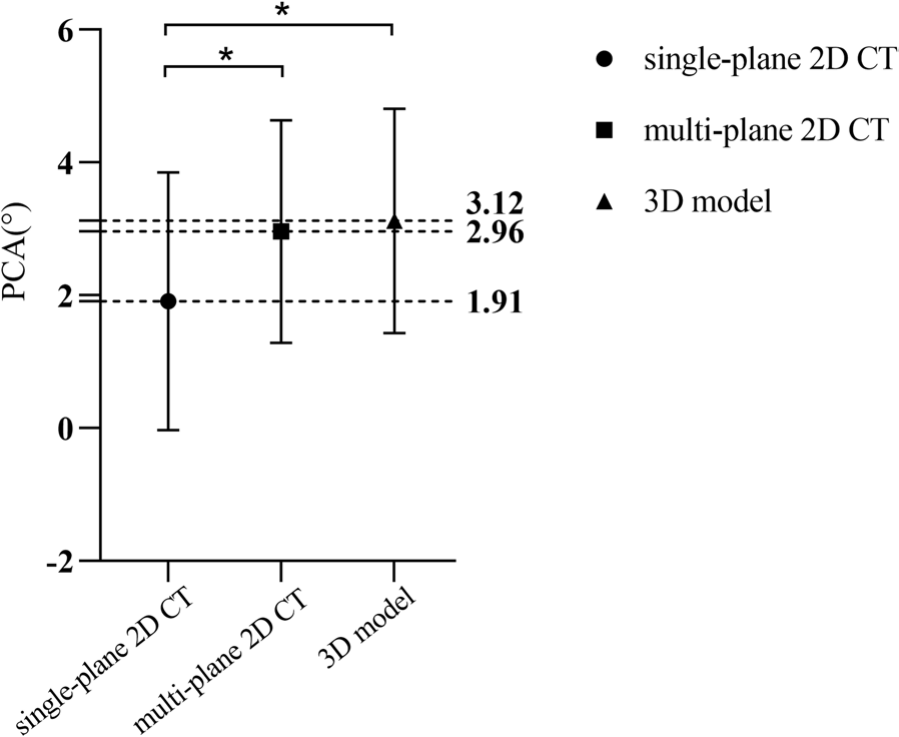
Comparison of PCA measurements among the three different methods. * indicates a statistically significant difference

The PCA measurements in single-plane 2D CT were 1.91 ± 1.94° (-4.00–6.11°), while those in multi-plane 2D CT were 2.96 ± 1.68° (-2.16–6.07°), and in 3D models were 3.12 ± 1.69° (-2.06–6.42°). There were significant differences in PCA measurements among the three methods (*P* < 0.001), with significantly smaller values in single-plane 2D CT than in multi-plane 2D CT or 3D models (*P* < 0.001, *P* < 0.001). However, there was no significant difference in PCA measurements between multi-plane 2D CT and 3D models (*P* = 0.103) (Fig. 5).

### Comparison of PCA measurement errors and outliers between two 2D CT measurement methods

**Fig. 6:**
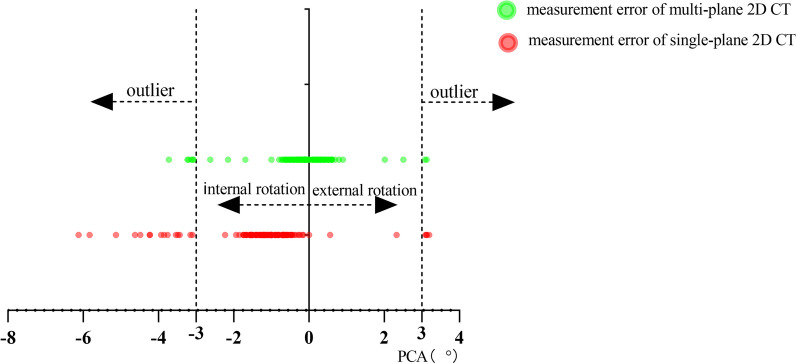
Distribution of PCA measurement errors and outliers in two 2D CT measurement methods

We evaluated the errors of PCA measured by two 2D CT methods. The measurement error of PCA in single-plane 2D CT was -1.22 ± 1.32°, while that in multi-plane 2D CT was -0.15 ± 0.91°. There was a significant difference in PCA measurement errors between the two 2D CT methods (*P* < 0.001). There were 19 (12.7%) outliers in PCA measurements with the single-plane 2D CT method, and 9 (6.0%) outliers in PCA measurements with the multi-plane 2D CT method, and the difference was statistically significant (*P* = 0.047) (Fig. 6).

### Reproducibility of PCA measurements using two 2D CT methods and a 3D model

#### Intra-observer reproducibility analysis of PCA measurements by different measurement methods

**Fig. 7:**
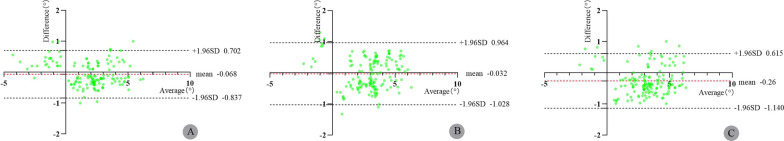
Bland–Altman plots of the differences between the two measurements of PCA by the same observer using three measurement methods. Figure A: the results of PCA measurements using single-plane 2D CT. Figure B: the results of PCA measurements using multi-plane 2D CT. Figure C: the results of PCA measurements using a 3D model

PCA measurements using single-plane 2D CT, multi-plane 2D CT, and 3D model were performed by the same measurer (KL) at different time points. The ICC values were 0.964 (0.951–0.974), 0.955 (0.938–0.967), and 0.965 (0.952–0.974), respectively, all of which were higher than 0.9, indicating a high level of consistency. The Bland–Altman plot showed that more than 95% of the differences between the two measurements of PCA by the same measurer in single-plane 2D CT, multi-plane 2D CT, and 3D model were within the limits of consistency (Fig. 7).

According to the evaluation criteria, the intra-observer PCA measurements using single-plane 2D CT, multi-plane 2D CT, and 3D model have demonstrated good reproducibility.

#### Inter-observer reproducibility analysis of PCA measurements by different measurement methods

**Fig. 8:**
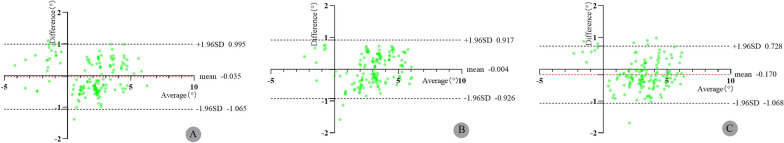
Bland–Altman plots of the differences between the two measurements of PCA using three measurement methods by different observers. Figure A: the results of PCA measurements using single-plane 2D CT. Figure B: the results of PCA measurements using multi-plane 2D CT. Figure C: the results of PCA measurements using a 3D model

Two assessors (KL, YDL) measured PCA using single-plane 2D CT, multi-plane 2D CT, and 3D model at the same time. The ICC values were 0.978 (0.970–0.984), 0.959 (0.944–0.970), and 0.964 (0.950–0.974), respectively, all greater than 0.9, indicating high consistency. The Bland–Altman plot revealed that more than 95% of the differences between the two measurements of PCA in single-plane 2D CT, multi-plane 2D CT, and 3D model by the two observers were within the limits of consistency (Fig. 8).

According to the evaluation criteria, the inter-observer PCA measurements using single-plane 2D CT, multi-plane 2D CT, and 3D model have demonstrated good reproducibility.

## Discussion

In this study, we utilized two 2D CT methods to measure PCA and compared the results with the reference values of PCA measured using a 3D model to evaluate their accuracy and reproducibility. The results revealed that all three measurement methods demonstrated good reproducibility for PCA measurements, both intra- and inter-observer. Moreover, the error of PCA measured by multi-plane 2D CT was significantly smaller than that of single-plane CT, and fewer outliers were observed.

The rotational alignment of the femoral component is a critical factor that affects the clinical outcome of TKA [6], and many studies consider sTEA as the gold standard for positioning the rotational alignment of the femoral component [19, 21, 26]. PCL is more easily identifiable intraoperatively than sTEA, and sTEA is usually externally rotated by approximately 3° relative to PCL [27]. Therefore, it is now routinely used to position the femoral component rotation by 3° externally relative to the PCL. This is consistent with our measurement results, where the PCA measured in the 3D model was 3.12 ± 1.69°. However, there is significant variability in PCA due to individual differences and factors such as age, race, and degree of osteoarthritis [13, 28, 29]. In this study, the range of PCA measurements in the 3D model was (-2.06°-6.42°), while Won et al. [30] reported a range of (1.2°-5.4°) in their measurement of PCA. Griffin et al. [31] measured PCA variances ranging from (0°-10°) in knees with combined osteoarthritis, and Koh et al. [32] measured PCA variances ranging from (-0.4°-7.3°) in a 3D femoral model reconstructed using MRI data. Therefore, some surgeons recommend preoperative planning with 2D CT to obtain individualized PCA for each patient.

Axial 2D CT images of the femur have been used for preoperative measurement of PCA in TKA, with the most obvious single-plane CT slice of sTEA commonly used to measure PCA [33]. While this 2D CT method of measuring PCA is relatively accurate, the four anatomical landmarks used to locate sTEA and PCL may not always be on the same CT slice, this approximate method of measuring PCA in a single-plane CT slice may lead to significant errors [34]. The accuracy of measuring PCA using a 3D femur model reconstructed from CT data has been recognized by many studies [19]. However, this method is complex and requires additional auxiliary techniques, making it less practical in certain clinical settings. In this study, it took approximately 3 min to perform a PCA measurement using single-plane 2D CT, 8 min using multi-plane 2D CT, and nearly 35 min using a 3D model. Therefore, there is a need to explore a simple, accurate, and reliable 2D PCA measurement method, that can be easily implemented in clinical practice.

In this study, the PCA measured using single-plane 2D CT was 1.91 ± 1.94°, significantly lower than that measured using a 3D model (3.12 ± 1.69°), with 12.7% of measurement errors exceeding 3°. Park et al.^[12]^compared the difference in measuring PCA between single-plane 2D CT slices and 3D models in a study that included 68 knee joints, and the results showed that the PCA measured using single-plane 2D CT slices differed from the reference value measured using 3D models by approximately 1°, with 9% of measurement errors exceeding 3°. Okamoto et al.^[20]^measured the PCA using single-plane 2D CT and 3D models in 75 knee joints before surgery, and the results showed that the measurements using single-plane 2D CT[2.3° (− 2.5–8.6°)] were significantly lower than those using 3D models[3.0°(− 2.0–7.5°)], with 13% of measurement errors exceeding 3°. These results are consistent with our study, indicating significant errors in measuring PCA using single-plane 2D CT. Considering the limitations of single-plane 2D CT, we innovatively used a multi-plane 2D CT method to measure PCA. First, we determined the most apparent single-plane 2D CT slices for each anatomical landmark used to locate sTEA and PCL and marked them on each plane of slice. Finally, we measured PCA in the same CT slice containing all anatomical landmarks. The results showed that there was no significant difference in PCA measurements between multi-plane 2D CT (2.96 ± 1.68°) and 3D models (3.12 ± 1.69°). The measurement errors were significantly smaller in multi-plane 2D CT (-0.15 ± 0.91°) compared to single-plane 2D CT (-1.22 ± 1.32°), and the proportion of outliers was only 6.0%. The reason for these results may be related to the following factors: first, the identification of anatomical landmarks remains a major problem in measuring PCA using single-plane 2D CT, and it is difficult to ensure that all four anatomical landmarks are on the same plane, which leads to errors in the localized PCL and sTEA. Clearly, this is influenced by the slice thickness, so the measured PCA is only an approximation [21, 35]; second, patients with knee osteoarthritis often have knee flexion and varus deformity, and it is difficult to maintain a strict body posture during CT scanning, making it challenging to ensure that the CT slice is perpendicular to the mechanical axis of the femur, the located sTEA and PCL are not on the transverse plane of the femoral mechanical axis, which can increase measurement errors when measuring PCA using single-plane 2D CT [20]. Multiple CT scan planes can locate relatively reliable anatomical landmarks, reducing these influences. Therefore, multi-plane 2D CT measurements have smaller errors than single-plane 2D CT measurements. Of course, measuring PCA on the surface of the reconstructed 3D femur model can more simply and accurately locate bony anatomical landmarks, which can well circumvent these limitations [21].

We also noted that the PCA measured in the single-plane 2D CT in this study differed from that measured in the multi-plane 2D CT and 3D model while the PCA measurements in the single-plane 2D CT were small, which may lead to excessive internal rotation of the femoral component and subsequent patellar maltracking and related complications [36]. Park et al. [12] also reported smaller PCA measurements in single-plane 2D CT as compared to 3D models, with 90% of outliers being due to excessive internal rotation of sTEA relative to PCL. We believe that this could be attributed to the direction of leg positioning during CT scanning. When there is varus or valgus deformity of the knee joint, the direction of CT scanning relative to the mechanical axis of the femur changes, causing the plane of CT slices to tilt. In a study comparing the differences in PCA measurement between single-plane 2D CT and 3D models, Okamoto et al. [20] found that when there is varus deformity of the knee joint, the medial femoral condyle is smaller in the CT slice, resulting in PCL being located more external rotation. However, the position of the sTEA does not show a significant change. Considering that most cases of knee osteoarthritis are accompanied by varus deformity, this could explain why the PCA values measured in single-2D CT slice are relatively smaller. However, when using multi-plane CT slices to locate PCL, the lowest points of the medial and lateral posterior condyles in multiple planes can be selected directly, which is not affected by the changing morphology of the medial femoral condyle in the slices. In clinical practice, it is difficult to ensure that the direction of CT scanning is parallel to the mechanical axis of the femur, so surgeons should be mindful of this issue.

In this study, the measurements of PCA in single-plane and multi-plane 2D CT as well as 3D models demonstrated high repeatability. The repeatability of PCA measurement in 3D models has been verified by many studies [21, 33]. However, some studies have reported poor repeatability when using single-plane 2D CT. For example, Hirschma et al. [21] reported ICC values of 0.63/0.32 for intra- and inter-observer consistency of PCA measurement, while Konigsberg et al. [33] reported ICC values of 0.386/0.606 for intra- and inter-observer consistency. In contrast, our study found that PCA measurement in single-plane 2D CT had good repeatability (0.96/0.98), which was consistent with the results of Park et al. [12]. Analyzing the differences, we found that both Park et al.'s study and our study excluded knee joints with unidentifiable medial epicondylar sulcus (type III morphology [37]) in the CT slices during PCA measurement. In contrast, the primary reason for the low repeatability observed in single-plane CT slice was the difficulty in locating bony landmarks [20]. Therefore, in our study, the anatomical landmarks of sTEA in 2D CT slices were relatively easy to identify and locate, resulting in high repeatability of PCA measurements in single-plane CT. In addition, when using multi-plane CT to measure PCA, the landmarks can be located at the most prominent planes for each anatomical landmark. In 3D model, the landmarks can be located directly on the surface of the femur under visual inspection. Both methods can achieve good visualization of the anatomical landmarks and reliable positioning of the bony landmarks, resulting in better repeatability of PCA measurements. Furthermore, the Bland–Altman plot showed that the mean difference of the intra-observer as well as inter-observer results of PCA measurements using the 3D model was larger (more deviated from 0) compared to that using single-plane and multi-plane 2D CT, and we analyzed that this might be related to the constructed 3D coordinate system because the process of constructing a 3D coordinate system is intricate, but the effect caused by a particular step needs to be further investigated, whereas the PCA measurements is relatively easy to perform in single-plane and multi-plane 2D CT.

Our study also has the following limitations: (1) The 3D model of the femur may not be entirely consistent with the actual anatomical structure, especially due to the surface smoothing operation performed on the femur model [38]; (2) Our results may not be applicable to all TKA cases, as preoperative CT is not routinely performed, and knee joints with unidentifiable medial epicondylar sulcus (type III morphology) were not analyzed in our study. Additionally, the risk of radiation exposure and economic costs should be considered; (3) CT slices and 3D CT models do not reflect the status of the femoral posterior condylar cartilage, and MRI-based measurements may be more accurate, as MRI takes into account the thickness of the residual cartilage in knee osteoarthritis; (4) While Mimics software is crucial for 3D printing technology and boasts a broad spectrum of clinical applications, there are instances where certain hospitals may lack access to this technology, and some doctors may not have mastered the use of Mimics software yet.; (5) Finally, our study did not include postoperative radiological parameters of the femoral component, which could further validate the effectiveness of preoperative planning and prove our conclusions.

## Conclusion

Preoperative 2D CT and 3D model measurements of PCA have good reliability in TKA. Multi-plane 2D CT has higher accuracy and fewer outliers in measuring PCA compared to single-plane 2D CT. Considering the limitations of intraoperative sTEA positioning, we recommend using multi-plane 2D CT to measure PCA preoperatively to assist in intraoperative positioning of the femoral component and improve the accuracy of the rotational alignment of the femoral component.

## Data Availability

The corresponding author can provide the datasets generated and analyzed during the current study upon reasonable request.
